# Biofilm Formation of Multidrug-Resistant MRSA Strains Isolated from Different Types of Human Infections

**DOI:** 10.3390/pathogens10080970

**Published:** 2021-07-30

**Authors:** Vanessa Silva, Luciana Almeida, Vânia Gaio, Nuno Cerca, Vera Manageiro, Manuela Caniça, José L. Capelo, Gilberto Igrejas, Patrícia Poeta

**Affiliations:** 1 Microbiology and Antibiotic Resistance Team (MicroART), Department of Veterinary Sciences, University of Trás-os-Montes and Alto Douro (UTAD), 5000-801 Vila Real, Portugal; vanessasilva@utad.pt; 2 Department of Genetics and Biotechnology, University of Trás-os-Montes and Alto Douro, 5000-801 Vila Real, Portugal; gigrejas@utad.pt; 3 Functional Genomics and Proteomics Unit, University of Trás-os-Montes and Alto Douro (UTAD), 5000-801 Vila Real, Portugal; 4 Associated Laboratory for Green Chemistry (LAQV-REQUIMTE), University NOVA of Lisboa, 2825-466 Lisboa, Portugal; 5 Veterinary and Animal Research Centre, Associate Laboratory for Animal and Veterinary Science (AL4AnimalS), University of Trás-os-Montes and Alto Douro (UTAD), 5000-801 Vila Real, Portugal; 6 Centre of Biological Engineering (CEB), Laboratory of Research in Biofilms Rosário Oliveira (LIBRO), Campus de Gualtar, University of Minho, 4710-057 Braga, Portugal; lucianamayara15@hotmail.com (L.A.); vaniagaio@ceb.uminho.pt (V.G.); nunocerca@ceb.uminho.pt (N.C.); 7 National Reference Laboratory of Antibiotic Resistances and Healthcare Associated Infections (NRL-AMR/HAI), Department of Infectious Diseases, National Institute of Health Dr Ricardo Jorge, Av. Padre Cruz, 1649-016 Lisbon, Portugal; vera.manageiro@insa.min-saude.pt (V.M.); manuela.canica@insa.min-saude.pt (M.C.); 8 Centre for the Studies of Animal Science, Institute of Agrarian and Agri-Food Sciences and Technologies, Oporto University, 4051-401 Oporto, Portugal; 9 BIOSCOPE Group, LAQV@REQUIMTE, Chemistry Department, Faculty of Science and Technology, NOVA University of Lisbon, 2825-466 Almada, Portugal; jlcm@fct.unl.pt; 10 Proteomass Scientific Society, 2825-466 Costa de Caparica, Portugal

**Keywords:** MRSA, human infections, biofilms, biofilm-related genes

## Abstract

Methicillin-resistant *Staphylococcus aureus* (MRSA) is one of the main pathogens causing chronic infections, mainly due to its capacity to form biofilms. However, the mechanisms underlying the biofilm formation of MRSA strains from different types of human infections are not fully understood. MRSA strains isolated from distinct human infections were characterized aiming to determine their biofilm-forming capacity, the biofilm resistance to conventional antibiotics and the prevalence of biofilm-related genes, including, *icaA*, *icaB*, *icaC*, *icaD*, *fnbA*, *fnbB*, *clfA*, *clfB*, *cna*, *eno*, *ebpS*, *fib* and *bbp*. Eighty-three clinical MRSA strains recovered from bacteremia episodes, osteomyelitis and diabetic foot ulcers were used. The biofilm-forming capacity was evaluated by the microtiter biofilm assay and the biofilm structure was analyzed via confocal scanning laser microscopy. The antimicrobial susceptibility of 24-h-old biofilms was assessed against three antibiotics and the biomass reduction was measured. The metabolic activity of biofilms was evaluated by the XTT assay. The presence of biofilm-related genes was investigated by whole-genome sequencing and by PCR. Despite different intensities, all strains showed the capacity to form biofilms. Most strains had also a large number of biofilm-related genes. However, strains isolated from osteomyelitis showed a lower capacity to form biofilms and also a lower prevalence of biofilm-associated genes. There was a significant reduction in the biofilm biomass of some strains tested against antibiotics. Our results provide important information on the biofilm-forming capacity of clinical MRSA strains, which may be essential to understand the influence of different types of infections on biofilm production and chronic infections.

## 1. Introduction

Methicillin-resistant *Staphylococcus aureus* (MRSA) is a major cause of community- and hospital-associated infections. MRSA can cause mild infections often associated with skin or soft tissue [1]; however, it can cause more severe infections such as pneumonia, osteomyelitis, cerebral abscess and sepsis, resulting in high rates of morbidity, high economic burden and possible mortality [2]. Since MRSA is one of the main causes of persistent human infections and its infections continue to be a major concern globally, MRSA was categorized as a high-priority pathogen by the World Health Organization (WHO) in 2017 [3].

MRSA infections are difficult to eradicate since these strains are often multidrug-resistant and the effectiveness of conventional antibiotics is compromised [4]. Overall, in the European Union (EU) and the European Economic Area (EEA), MRSA accounts for approximately 150,000 of hospital-associated infections each year, resulting in more than 7000 deaths and a socioeconomic burden of EUR 380 million annually [5,6]. The incidence of MRSA infections in the EU and EEA varies significantly between the north and south, with southern countries reporting above-median MRSA proportions [7]. In fact, although a decrease in the weighted proportion of MRSA among *S. aureus* isolates from 2007 to 2015 was reported, a study by Cassini et al. (2019) showed that the estimated incidence of MRSA infections actually increased 1.28-fold [6]. Moreover, although some countries, including Portugal and Romania, reported a decrease in MRSA infections, these countries continue to experience a substantial burden due to MRSA infections, with prevalence levels above the European average [8].

Compounding the problem even further is the fact that MRSA can form biofilms on biotic and abiotic surfaces [9]. For many decades, staphylococci have been recognized as the most frequent cause of biofilm-associated infections [8,10]. However, most research has focused on bacteria growing in planktonic cultures, while antibiotics were originally developed to target individual bacterial cells [11]. Nevertheless, it is clear that bacteria prefer to grow as sessile communities [11]. It has been suggested that biofilms are responsible for nearly 80% of all human infections and one of their most critical features is their high level of resistance to antibiotics, host immune defenses, disinfectants and environmental stress [10,11]. Biofilms are usually associated with medical devices such as catheters, mechanical heart valves, joint prostheses and orthopedic devices but can also be associated with other infections, such as endocarditis and osteomyelitis [12,13]. Biofilms are more resistant to antibiotics than planktonic cells due to the multi-level protection conferred by the extracellular matrix (which hinders the penetration of antibiotics), altered metabolic states and growth rate [14,15]. Furthermore, the biofilm formation ability of MRSA strains, together with their often associated multidrug-resistance profile, enhances the overall resistance, resulting in chemotherapeutic failure [16]. In addition, the close proximity of bacterial cells within the biofilm promotes horizontal genetic transfer, conjugation and mobilization of antimicrobial resistance genes [9].

Biofilm formation is divided into at least three stages: initial attachment, biofilm maturation and dispersal [17]. The first stage is mediated mainly through the microbial surface components recognizing adhesive matrix molecules (MSCRAMMs) [18]. The maturation is characterized by the production of polysaccharide intercellular adhesin (PIA) synthetized by the products of the *icaADBC* operon [19,20]. The final step, dispersal, allows the recolonization of other available host sites [12]. Although some studies have reported the biofilm-forming capacity of MRSA strains, it is important to conduct more studies that will allow us to understand the mechanism underlying biofilm formation associated with different human infections, particularly studies that compare MRSA isolates from distinct infections [20,21,22]. Only a very limited number of studies have compared the biofilm-forming ability of MRSA strains with the type of human infection [14,22]. In fact, in Portugal, where the percentage of MRSA strains isolated from clinical infections is above the European average, as far as we know, no studies have been conducted regarding the comparison between the type of infection and the biofilm-forming ability of MRSA or *S. aureus* strains.

Therefore, we aimed to characterize the biofilm-forming ability of 83 MRSA strains isolated from different human infections, to investigate the prevalence of biofilm-related genes and to study the anti-biofilm efficacy of conventional antibiotics. Furthermore, we also performed the whole-genome sequencing (WGS) of two strains based on their biofilm-forming capacity.

## 2. Results

### 2.1. Biofilm Formation

**Figure 1:**
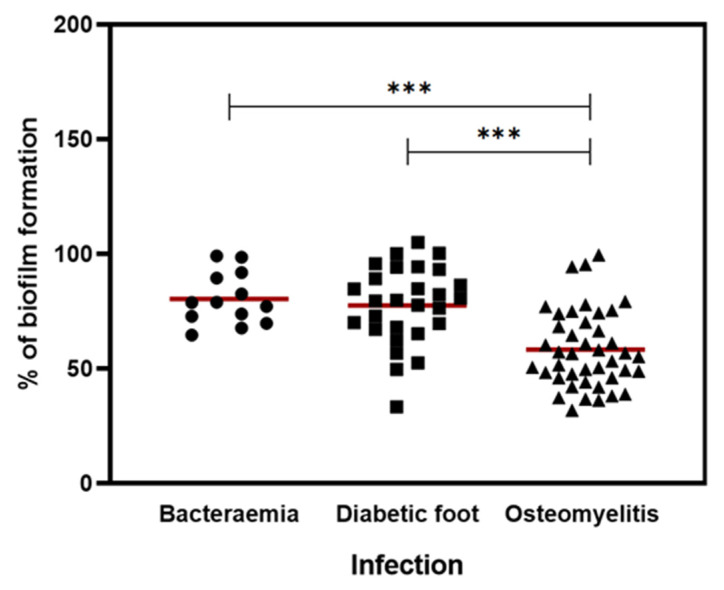
Ability of MRSA strains to form biofilm associated with the type of infection. Comparison of the biofilm formation capacity of clinical isolates from different infections, including bacteremia (n = 13), diabetic foot (n = 28) and osteomyelitis (n = 42). The symbols (●, ■, ▲) represent the average biomass of the biofilm formed in independent tests of the individual isolates tested for each type of infection. The red lines represent the average of biofilm biomass formed by all isolates of each type of infection. Statistical significance was determined using one-way analysis of variance (one-way ANOVA) followed by Tukey’s multiple comparison test. Significant differences are described with *** *p* < 0.001.

A microtiter plate assay was used to measure the biofilm production of 83 MRSA strains isolated from human infections, namely 13 from bacteremia episodes, 42 from osteomyelitis and 28 from diabetic foot ulcers. The results were normalized against *S. aureus* ATCC 25923 so that the comparison of results could be more reliable. The percentage of biofilm formation of each strain is shown in Figure 1. 

The percentage mean of biofilm formation for all isolates from bacteremia, diabetic foot infections and osteomyelitis was 80.5%, 77.6% and 58.3%, respectively. The biofilm production of osteomyelitis strains was significantly lower than the biofilm production of strains isolated from other infections (*p* < 0.001). Although the biofilm production of strains from bacteremia was higher than this, this difference was small and not statistically significant. MRSA strain O20, which belongs to the osteomyelitis group, was the weakest biofilm producer (31.9%), and the strain D2 was the strongest biofilm producer (105.2%) and belonged to the diabetic foot infection group. Both strains were analyzed by whole-genome sequencing.

### 2.2. Confocal Scanning Laser Microscopy (CLSM) Analysis

**Figure 2:**
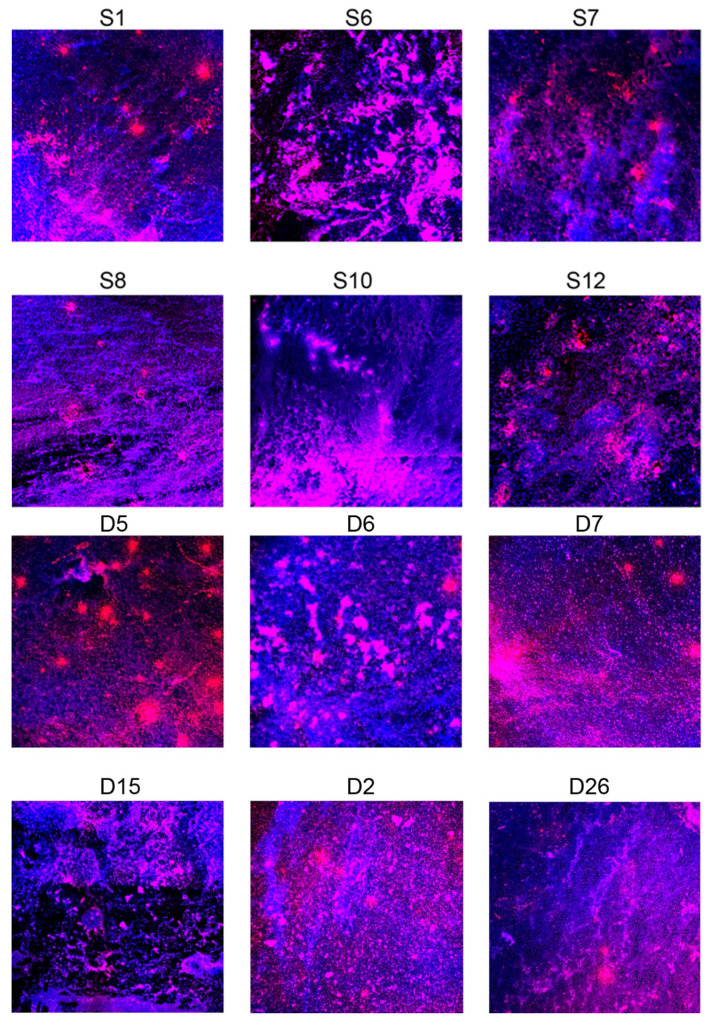

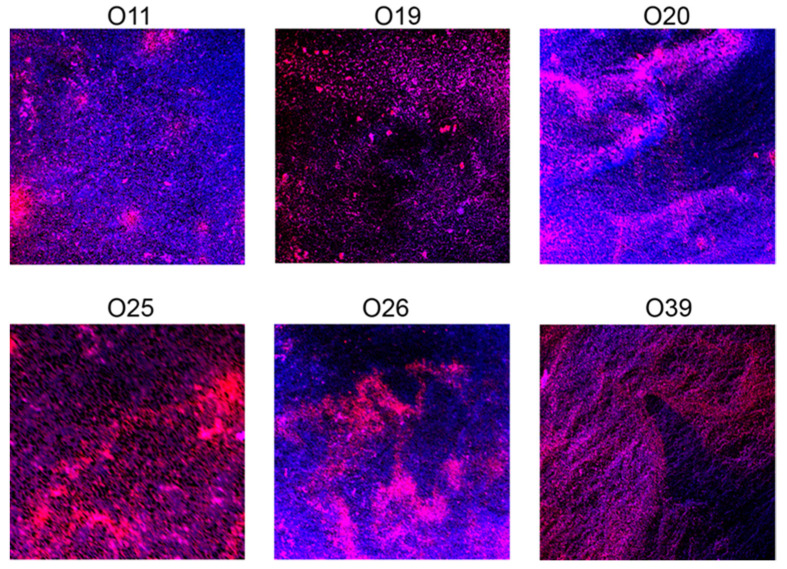
Scanning confocal laser microscope images of 24 h MRSA biofilms. (S: bacteremia; D: diabetic foot; O: osteomyelitis). The images represent the colocalization of both probes: diamidino-2-phenylindole (DAPI) probe (which binds to nucleic acids) is visualized in blue and the Tetramethylrodamine (WGA-TRITC) probe (which binds to polysaccharides) in red.

CLSM was used to visualize the overall morphology of MRSA biofilms. For CSLM analysis, 18 strains (6 of each type of infection) were carefully selected according to their biofilm-forming ability and the phenotypic antimicrobial resistance profiles of the planktonic cells. Strains from bacteremia, diabetic foot ulcers and osteomyelitis have been previously characterized regarding antimicrobial resistance, virulence and genetic lineages [1,23,24,25]. Figure 2 shows the images obtained by CLSM of each strain. Not surprisingly, strains recovered from osteomyelitis produced less clusters when compared with the images from bacteremia and diabetic foot strains.

### 2.3. WGS

To understand the genetic background of O20 and D2 clinical strains, WGS was performed. The analysis of the genome sequences yielded 273 and 212 contigs, respectively (from 200 to 127,245 bp, and from 203 to 137,189, respectively) (Table 1). The draft genome contained a total assembly length of 2,795,149 and 2,788,919 bp, respectively; the GC content was 32.7%.

In silico analysis revealed the presence of loci associated with virulence, disease and defense (Table 2). The virulence factor *sdrE*, which encodes for the serine-aspartate repeat protein and is involved in biofilm adhesion, was exclusively identified in the D2 strain. Sequences coding for functions related to mobile genetic elements were identified, which include different plasmid types found in these replicons as well as open reading frames (ORFs) associated with different insertion sequences.

The total number of determinants matching pathogenic families, which, according to PathogenFinder, includes, for instance, virulence factors, antibiotic resistance genes and mobile genetic elements, showed 97.8% and 98.3% certainty that both O20 and D2 strains were human pathogens, confirming the pathogenicity of these isolates. 

The bioinformatics analysis of the genetic relatedness was carried out regarding multilocus sequence typing (MLST), protein A gene (*spa*), accessory gene regulator (*agr*; which encodes for the two divergent transcripts RNAII and RNAIII) and staphylococcal cassette chromosome *mec* (SCC*mec*) typing. These two strains had completely different typing results. D2 was ascribed to ST105, SCC*mec* type II, *spa*-type t535 and *agr* II while the O20 isolate belonged to ST22, SCC*mec* type IV, *spa*-type t6966 and *agr* type IV. Studies have reported a relation between the biofilm-forming capacity of *S. aureus* and the molecular typing, showing that strains belonging to certain clonal lineages may have greater or lesser capacity to form biofilms [26,27]. 

### 2.4. Biofilm-Related Genes

**Figure 3:**
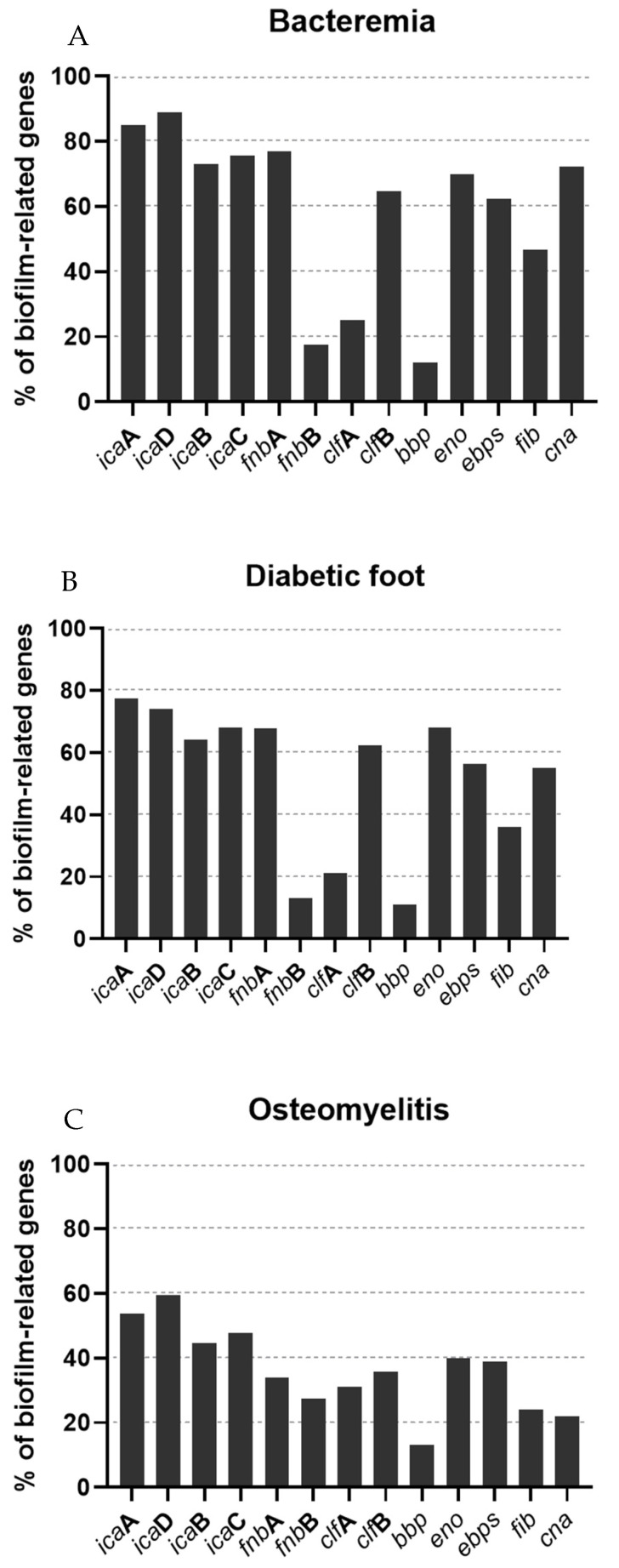
Percentage of biofilm-related genes associated with the type of infection. (**A**): Bacteremia; (**B**): Diabetic foot infection; (**C**): Osteomyelitis.

Since the variation of only one or two biofilm-related genes may induce more or less biofilm production, we studied the prevalence of 13 genes involved in biofilm production, *icaA, icaB, icaC, icaD, fnbA, fnbB*, *clfA, clfB, cna, eno*, *ebpS*, *fib* and *bbp,* performed by PCR. As shown in Figure 3, the most prevalent genes were the *ica* genes, followed by *eno*, detected among the isolates recovered from all types of infections. *icaA* (85%, 77.4% and 53.7% in bacteremia, diabetic foot and osteomyelitis isolates, respectively) and *icaD* (89.1%, 74.1% and 59.6 5 in bacteremia, diabetic foot and osteomyelitis isolates, respectively) were more prevalent than *icaB* (73%, 64.1%, 44.5% in bacteremia, diabetic foot and osteomyelitis isolates, respectively) or *icaC* (75.5%, 68% and 47.8% in bacteremia, diabetic foot and osteomyelitis isolates, respectively) in all strains. The *fnbB* gene was less frequently detected than *fnbA*, which had a prevalence similar to *icaB* and *icaC*. Unlike osteomyelitis strains, which had a higher prevalence of *clfA* than *clfB*, the *clfB* gene was more prevalent in strains from bacteremia and diabetic foot ulcers than *clfA*. The *eno* gene had also high frequencies among the isolates, being slightly more prevalent in diabetic foot isolates. The prevalence of *bbp* was almost identical in isolates from the three types of infections and it was the least frequent gene detected among the isolates. The *ebps* and *fib* genes were more prevalent in isolates from bacteriemia, followed by diabetic foot infections and osteomyelitis. Finally, the *cna* gene was detected in around 70% of bacteremia isolates, followed by 55% of diabetic foot infections and 22% of osteomyelitis isolates.

There is a significant difference of the total content of biofilm-related genes when comparing the total amount of biofilm-related genes within each type of infection (data not shown). The prevalence of biofilm-related genes in bacteremia isolates is statistically different from isolates of diabetic foot (*p* < 0.05), and the prevalence of these genes in bacteremia and diabetic foot isolates is also statistically different from osteomyelitis isolates (*p* < 0.001).

### 2.5. Antimicrobial Susceptibility of 24-h-Old Biofilms

**Figure 4:**
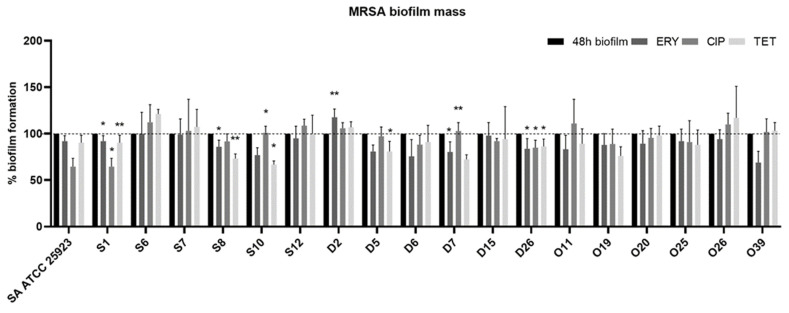
Effect of different antibiotics on the biofilm formation capacity of MRSA isolates. Quantification of biofilm biomass after an additional 24 h incubation in the presence of different antibiotics. The concentrations used for each antibiotic correspond to the peak serum concentration, with 4.5 mg/L for ciprofloxacin (CIP), 10.0 mg/L for erythromycin (ERY) and 16.0 mg/L for tetracycline (TET). Data are presented as mean ± standard deviation for three independent trials. Statistical significance was determined using one-way analysis of variance (one-way ANOVA) followed by Tukey’s multiple comparison test. Significant differences are described with * *p* < 0.05; ** *p* < 0.005. (S = bacteremia, D = diabetic foot, O = osteomyelitis).

In order to investigate the capacity of conventional antibiotics to reduce pre-established 24-h-old biofilms, the 18 strains (6 of each type of infection) analyzed by CSLM were used. The microtiter biofilm assay was used to determine the capacity of antibiotics to reduce the biofilm biomass. Results were normalized according to the 48-h-old biofilm mass recorded for each strain tested, which were grown without antimicrobial agents. After obtaining 24-h-old biofilms, the medium was replaced by fresh medium with the antibiotic. As shown in Figure 4, strains S1, S8, D7 and D26 had a significant decrease in biofilm mass when treated with erythromycin. On the contrary, in strain D2, there was a significant increase in biomass when treated with erythromycin (*p* < 0.05). Regarding the strains treated with ciprofloxacin, there was a significant decrease in biofilm mass in strains S1, S8 and D26 and a significant increase in strains S10 and D7. Strains S1, S8, S10, D5 and D26 suffered a significant biomass decrease when treated with tetracycline. Nevertheless, it seems that the phenotypic antimicrobial resistance does not influence the effect of antibiotics in biomass reduction since in both strains, S1 and D26, the biomass was reduced significatively with all antibiotics and these strains had resistance to ciprofloxacin and to the three antibiotics, respectively.

### 2.6. Effect of Antibiotics on Metabolic Activity

**Figure 5:**
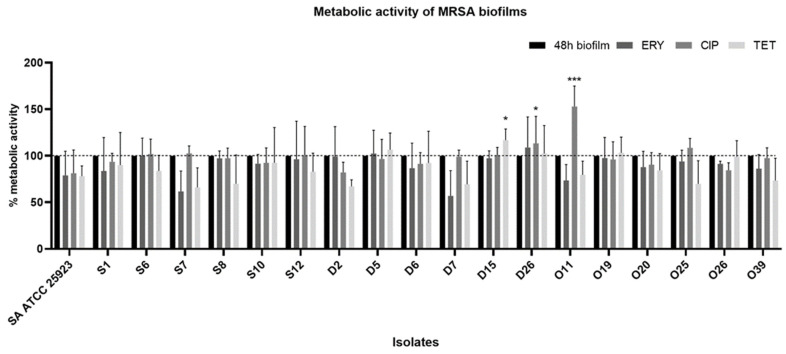
Metabolic activity of MRSA biofilms before and after exposure to antibiotics. The results are expressed as percentage of metabolic activity. The results were normalized according to the 48-h-old biofilm of each tested strain. Statistical significance was determined using one-way analysis of variance (one-way ANOVA) followed by Tukey’s multiple comparison test. Significant differences are described with * *p* < 0.05; *** *p* < 0.001. ERY: erythromycin, CIP: ciprofloxacin, TET: tetracycline.

The XTT assay was used to evaluate the biofilms’ metabolic activity after exposure to antibiotics. The effects of the different antibiotics on the metabolic activity of biofilms are summarized in Figure 5. The results were normalized according to the 48-h-old biofilm of each tested strain (which were grown without antimicrobial agents). Overall, it seems that there is not much difference between biofilms exposed and not exposed to antibiotics. In fact, there was an increase in the metabolic activity of the D15 strain when treated with tetracycline (*p* < 0.05) and of strains D26 (*p* < 0.05) and O11 (*p* < 0.005) when treated with ciprofloxacin.

## 3. Discussion

The biofilm-forming capacity of bacterial strains is a trait highly associated with bacterial persistence and virulence [26]. Furthermore, chronic bacterial infections are linked to the formation of biofilms [26]. Biofilm-producing capacity is closely related to clinical *S. aureus* strains, genetic lineages, multidrug-resistance profiles and highly virulent strains [26]. As far as we are aware, no data exist regarding MRSA and biofilm association with strains isolated in Portugal. Herein, we characterize the biofilm formation ability of several MRSA Portuguese isolates obtained from bacteremia episodes, skin wounds (diabetic foot) and osteomyelitis.

All MRSA strains had the ability to adhere to the microplate and form biofilms (Figure 1). MRSA from bacteremia and diabetic foot produced more biofilm biomass than strains from osteomyelitis. Other studies had similar results, with isolates from blood producing more biofilm mass than isolates from other infections, such as skin lesions, urinary tract infections and sputum [14,28]. However, there are some contradictory results since it was also shown that some MRSA blood isolates display low-level biofilm formation [26,29]. This may be due to the fact that biofilm formation capacity is also influenced by other factors, such as the virulence genes carried by MRSA strains and the clonal lineages [30,31]. The biofilm-forming ability of strains from bacteremia and diabetic foot ulcers had similar results and the difference was not statistically significant. Isolates from the skin have proven to have a high ability to form biofilms, which may be due to the fact that the biofilm’s mode of growth may increase protection against topical antimicrobial agents routinely used in skin wounds [29]. The presence of MRSA strains with a high capacity to form biofilms in blood is a concern since MRSA may colonize other sites and generate secondary infections, such as infective endocarditis, septic arthritis and osteomyelitis [32]. 

In our study, 18 strains (6 from each type of infection) were carefully selected based on their antimicrobial resistance profiles and biofilm-forming capacity to perform the CLSM analysis (Figure 2). After the visualization of the biofilms with CLSM, it was shown that the selected strains were able to form compact biofilms on polystyrene after 24 h of incubation, as reported in other studies [5,31]. However, as expected, and in accordance with the biofilm-forming capacity results, strains recovered from osteomyelitis produced less clusters than strains from the other two types of infection.

It has been shown that biofilm formation is a dynamic process influenced by biofilm-related genes and regulatory reactions [33]. Therefore, WGS of the most biomass-producing strain (D2) and the strain that produced the least biomass (O20) was performed in order to verify the differences related to virulence factors and biofilm genes (Table 2). Overall, both strains harbored almost the same genes and similar pathogenicity-related genes. Nevertheless, novel genes present only in one strain, particularly the strong biofilm-producing strain, could contribute to the difference in biofilm-forming capability. The large difference in biofilm production between strains D2 and O20 may be related to the fact that the *sdrE* and *icaB* genes were exclusively identified in the D2 strain. SdrE is a surface protein that belongs to the Sdr protein subfamily, and it is known to be essential for biofilm growth via homophilic interaction between the N2 subdomains likely occurring on neighboring bacteria [34,35]. The IcaB protein is encoded by the *icaB* of the *icaADBC* operon. This protein is the deacetylase responsible for the deacetylation of poly-N-acetylglucosamine, which is essential for biofilm formation [28,36]. The absence of IcaB may be responsible for the weak biofilm production since this leads to the synthesis of poly-N-acetylglucosamine with deacetylation, which is less efficient in binding to the bacterial cell surface, leading to a reduction in biofilm formation [28,36].

We studied the prevalence of 12 genes involved in biofilm production. The most frequently detected genes were *icaA*, *icaD*, *fnbA* and *eno* (Figure 3). *icaA* and *icaD* are necessary factors for intercellular adhesion and forming a bacterial multilayer in biofilm production and are associated with both slime and biofilm formation in *S. aureus* [22]. Strains from osteomyelitis carried less biofilm-related genes when compared to strains from bacteremia and diabetic foot infections. Biofilm formation is highly associated with the expression of *ica* genes and a study has shown that *ica*-positive MRSA biofilms create thicker biofilms with a more compact architecture than *ica*-negative isolates using CLSM analysis, which is accordance with our results [37]. Other studies had *ica* genes frequencies in clinical MRSA isolates similar to ours [26,38]. In all of our isolates, the *fnbB* gene was less frequently detected than *fnbA*. Cha et al. (2013) reported the *fnbB* gene as the least predominant in biofilm-producing strains isolated from blood [26]. *fnbA* and *fnbB* encode for two adhesins, FnbA and FnbB, which are very relevant for the virulence action of MRSA strains in human hosts. Both adhesins play a significant role in tissue colonization in osteomyelitis and septic arthritis, and in dwelling medical devices, which may explain the higher frequency of the *fnbB* gene in osteomyelitis isolates [39].

*clfA* is the major *staphylococcal* fibrinogen-binding protein and it was also more frequent in osteomyelitis isolates [18]. In accordance with our results, a study conducted by Yu et al. examined the biofilm formation capacity and the prevalence of biofilm-related genes of 137 orthopedic *S. aureus* isolates and also reported a high prevalence of this gene in *S. aureus* isolates [40]. Wang et al. reported that *ClfA* impairs orthopedic implant-associated hematogenous *S. aureus* infection in a mouse model and that neutralizing antibodies against *ClfA* inhibited *S. aureus* biofilm formation [41]. On the contrary, the *clfB* gene was more prevalent in strains from bacteremia and diabetic foot ulcers than *clfA*. The clumping factor B (*ClfB*) was identified as a major determinant of nasal colonization [42]. Nevertheless, Wang et al. showed that both *clfA* and *clfB* were present in all isolates recovered from bone/joint infections, skin/soft tissue infections and catheter-related bacteremia [43].

The prevalence of the *eno* gene is in accordance with Szczuka et al., who found a higher prevalence of the *eno* gene in isolates from wounds than in isolates from bacteremia [37]. Regarding the *bbp* gene, similarly to our results, other studies have reported an absence or a low prevalence of the *bbp* gene in *S. aureus* isolates [18,44]. In our study, the *ebps* and *fib* genes were more prevalent in isolates from bacteremia. *ebps* was the most prevalent in the study by Bride et al., conducted with *S. aureus* strains isolated from healthcare-associated infections from various sources including blood [45]. Regarding the *fib* gene, other studies conducted with MRSA and *S. aureus* strains isolated from blood and wounds had a higher prevalence of this gene when compared to our study [46,47]. Nevertheless, a study conducted in MRSA from burn patients reported a prevalence of *fib* similar to ours [48]. Finally, *cna* was detected in most of the bacteremia isolates, followed by diabetic foot infections. Yu et al. studied the prevalence of several virulence genes, including biofilm-related genes, in *S. aureus* isolated from bloodstream infections and reported a lower prevalence (50.6%) of *cna* [49]. In contrast, G haznavi-Rad et al. reported a *cna* prevalence of 98.7% in *S. aureus* isolated from clinical samples.

It has been shown that bacteria within biofilms are up to 1000-fold more resistant to antibiotics than planktonic bacteria [50]. The 18 strains (6 from each type of infection) selected for CLSM analysis were also used to assess the antimicrobial susceptibility of biofilms and to perform the XTT assay. Several studies have been conducted regarding the MBIC of conventional antibiotics [51,52,53,54]. However, MBIC concentrations are often several fold higher than the peak serum concentration, and as such, these studies do not provide results applicable in the in vivo situation. To assess biofilm tolerance to clinically relevant antibiotics, 24 h-old biofilms from the selected isolates were exposed erythromycin, ciprofloxacin and tetracycline (Figure 4). Although strains S8 and D26 were resistant to erythromycin, there was a significant decrease in biofilm biomass when treated with this antibiotic. Studies have shown a positive effect of macrolides on the reduction in biofilm mass [55,56]. Furthermore, a study by Mottola et al. reported that the minimum biofilm inhibitory concentration (MBIC) for erythromycin against *S. aureus* recovered from diabetic foot ulcers increased around four times in comparison with the values for the minimum inhibitory concentration (MIC), from 0.12 –>256 μg/mL to 0.5 – >256 μg/mL [51]. Parra-Ruiz et al. (2012) showed that low levels of macrolides can inhibit the biofilm formation process of *S. aureus* [56]. In contrast, another study demonstrated that erythromycin can stimulate the expression of *ica* genes, leading to an increase in biofilm formation [57].

Ciprofloxacin led to a reduction in the biofilm on a smaller number of isolates, which might be explained by the fact that 13 out of 18 isolates showed phenotypic resistance to ciprofloxacin when tested by the Kirby–Bauer disc diffusion method, as reported elsewhere [1,23,24,25]. The ciprofloxacin concentration used in our study was 10 mg/L, which corresponds to the peak serum concentration. Studies have reported that the MBIC of ciprofloxacin for *S. aureus*, either resistant or susceptible to ciprofloxacin, varies from 4 to 128 mg/L [52,58]. Only three isolates were resistant to tetracycline, yet this antibiotic had a positive effect on the reduction of biofilm mass in five isolates. Indeed, it has been shown that the tetracycline group of antibiotics is quite effective in eradicating MRSA biofilms [53]. Furthermore, other studies on *S. epidermidis* biofilms demonstrated that antibiotics that target protein or RNA syntheses have higher efficiency than antibiotics that target cell wall synthesis, and this has been associated with the lower growth rate of cells within biofilms [17,59,60,61]. Studies have reported that tetracycline suppresses the localization of the autolysin Atl, which plays an important role in the initial attachment of the biofilm [62,63]. Although MRSA strains from osteomyelitis presented the lowest biofilm formation, they were also the most resistant to the action of antibiotics on the 24-h-old biofilms.

The crystal violet (CV) assay stains all cells and quantifies the matrix of both living and dead cells [33]. Therefore, after exposure to antibiotics, the metabolic activity of biofilm cells was evaluated using the XTT assay. Overall, none of the antibiotics used were able to significantly reduce the metabolic activity of the strains (Figure 5). In contrast, the metabolic activity was not significantly increased in 3 out of 18 strains. Although the XTT method has the advantage of being a fast assay, it also has some limitations, one of them being the low sensitivity [54]. In one study, the treatment of pre-established biofilms with chloramphenicol alone or together with antimicrobial peptides did not produce a substantial change in biofilm viability after 24 h of treatment [64]. Another study using natural products against *S. aureus* biofilms did not show a reduction or showed a small reduction in the metabolic activity after 24 h of exposure for some natural products [65]. 

In our study, strain D26 presented a decrease in biofilm biomass and an increase in the metabolic activity after 24 h of exposure to antibiotics. Indeed, it is not possible to determine whether there are actually more living cells or if the cells are increasing their metabolism in an attempt to resist the external pressure caused by antibiotics. Another possible explanation is that the strains might have been at the proliferative stage, with a lesser extracellular matrix. Xu et al. (2016) compared the CV and XTT assays on *S. aureus* biofilm quantification and reported that some strains seemed to be strong biofilm producers according to the CV results, but these strains were found to have weaker metabolic activity than strains that produced less biofilm and vice versa [33]. 

## 4. Materials and Methods

### 4.1. Bacterial Strains and Growth Conditions

In this study, 83 clinical MRSA strains recovered from bacteremia episodes (n = 13), osteomyelitis (n = 42) and diabetic foot ulcers (n = 28) were used. These strains have been previously characterized regarding the antimicrobial resistance, virulence and genetic lineages [1,23,24,25]. The isolates were obtained under the approval of the Ethics Committee of the University of Trás-os-Montes and Alto Douro (CE-UTAD), Vila Real, Portugal. *S. aureus* ATCC^®^ 25923 (clinical isolate) was used as a positive control due to its excellent biofilm formation capacity. The isolates were cryopreserved at −80 °C in Tryptic Soy Broth solution (TSB; Liofilchem, Teramo, Italy) with 30% (*v*/*v*) glycerol (Panreac, Barcelona, Spain).

### 4.2. Biofilm Formation Assay

The biofilm formation assay was performed as previously described by Oniciuc et al. (2016), with some modifications [66]. Briefly, a few colonies were transferred from fresh cultures to 10 mL volume Erlenmeyer flasks with 2.5 mL of TSB and incubated at 37 °C for 16 ± 1 h with continuous shaking at 120 rpm (ES-20 Shaker-Incubator, BioSan, Riga, Latvia). The bacterial suspension was adjusted to an optical density of 0.25 ± 0.05 at OD640 nm, corresponding to a concentration of 2 × 10^8^ colony-forming units (Biochrom; EZ Read 800 Plus). Then, 198 µL of TSB supplemented with 3% (*w*/*v*) NaCl and 2 µL of bacterial suspension of different isolates was added to each well of a 96-well flat-bottom microplate (Orange Scientific, Braine-l’Alleud, Belgium). *S. aureus* ATCC^®^ 25923 was included in all plates as a positive control. Fresh medium without bacterial inoculum was used as a negative control. The plates were incubated at 37 °C for 24 h. All experiments had seven technical replicates and were performed in triplicate.

#### Biofilm Biomass Quantification

Biofilm biomass was quantified using the CV staining method as previously described by Peeters et al. (2008), with some modifications [67]. After incubation, the bacterial cells in suspension were removed and the plates were washed twice with 200 µL of distilled water. The plates were then allowed to dry at room temperature for 2 h. Then, 100 µL of methanol (Fisher Scientific, Leicestershire, UK) was added to each well and incubated for 15 min to fix the biofilm. Methanol was removed, the plates were allowed to dry at room temperature for 10 min, and 100 µL of CV at 1% (*v*/*v*) (Acros Organics, NJ, USA) was added to each well. After 5 min, the CV solution was removed, and the excess dye was removed by washing the plates twice with 200 µL of distilled water. Then, 100 µL 33% (*v*/*v*) of acetic acid (Fisher Scientific) was added to solubilize the CV and the absorbance was measured at 595 nm using a microplate reader (Biochrom, EZ Read 800 Plus, Cambridge, UK). In order to standardize the results, the biofilm formation of each isolate was normalized according to the positive control strain, *S. aureus* ATCC^®^ 25923.

### 4.3. CLSM Analysis

Eighteen strains (6 of each type of infection) representative of the bacterial collection were used. This particular set of strains was chosen according to their biofilm-forming capacity and susceptibility to the tested antibiotics (Table 3): strains D5, D6 and O26 were sensitive to eryhtromycin, ciprofloxacin and tetracycline; strains S1, S7, S12, D7, O11 and O39 were resistant to ciprofloxacin; strains S6, S8, S10, D2, D15, O20 and O25 had resistance to both erythromycin and ciprofloxacin; strain D26 was resistant to the three antibiotics; and strain O19 showed resistance to both erythromycin and tetracycline [1,23,24,25]. Biofilm formation was performed as described in Section 2.2., but using 24-well plates (Orange Scientific, Braine-l’Alleud, Belgium). A 13 mm diameter sterile plastic coverslip (Nunc^®^ Thermomanox^®^ Plastic Coverslips, New York, NY, USA) was placed at the bottom of each well. Ten microliters of bacterial suspension was added to 990 µL of TSB with 3% NaCl and incubated at 37 °C for 24 h [68]. Then, culture medium was removed, and the biofilms were washed with 1 mL of 0.9% (*w*/*v*) NaCl solution. The plastic coverslips were transferred to microscopic slides and stained. All staining procedures were performed in the dark and according to the manufacturer’s instructions. A fluorescent probe of wheat germ agglutinin conjugated to WGA-TRITC (Invitrogen, Carlsbad, CA, USA) and DAPI fluorescent nuclear probe (Sigma-Aldrich, Saint Louis, MO, USA) were used to allow the staining of residues of N-acetyl-D-glucosamine (GlcNAc) and to allow visualization of the cells, respectively [69]. One hundred microliters of WGA-TRITC at a concentration of 10 µg/mL was added to each surface and incubated for 10 min. After incubation, the excess dye was removed and 100 µL of DAPI at a concentration of 100 µg/mL was added. After 5 min of incubation, the excess dye was removed. The fluorescence of the WGA-TRITC was detected using a laser with an excitation wavelength of 559 nm and an emission filter of 505–605 nm. DAPI fluorescence was detected using a laser with an excitation wavelength of 405 nm and an emission filter of 430–470 nm. Stained biofilms were visualized using the CLSM Olympus FluoView FV1000 (Olympus, Lisbon, Portugal) with a 10× objective and a 2× electronic magnification. Images of different regions of each surface were acquired with a resolution of 640 × 640 pixels. Two technical replicates were used to select representative images.

### 4.4. WGS

#### 4.4.1. Genomic DNA Preparation and WGS

Two strains were selected according to their biofilm production capacity, the most productive and the least productive (O20 and D2), to perform the WGS. Genomic DNA of O20 and D2 strains was extracted using the MagNa Pure 96 Instrument (Roche, Germany), and DNA quantification was performed by Qubit Fluorometric Quantitation (Life Technologies, Carlsbad, CA, USA), according to the manufacturer’s instructions. Libraries were prepared from 1 ng of genomic DNA using the Nextera XT DNA Sample Preparation Kit (Illumina, San Diego, CA, USA), according to the manufacturer’s instructions. WGS was performed using 150 bp paired-end reads on a MiSeq (Illumina, San Diego, CA, USA). 

#### 4.4.2. Genome Assembly and Annotation

Sequence reads were trimmed and filtered according to quality criteria, and assembled de novo using CLC genomics workbench version 10.0.1 (QIAGEN Aarhus, Denmark). The generated contigs were submitted for annotation in Prokka v.1.12 [70].

#### 4.4.3. Genomic Analysis

Downstream bioinformatic analyses were performed by means of online tools and databases available at the Center for Genomic Epidemiology (CGE) (www.genomicepidemiology.org, accessed on 15 January 2021) to investigate the presence of antimicrobial resistance genes (ResFinder 4.1, https://cge.cbs.dtu.dk/services, accessed on 15 January 2021), plasmids (PlasmidFinder 2.1) and pathogenicity determinants (PathogenFinder 1.1). In silico MLST was performed using MLST v.2, *spa* typing was predicted using spaTyper 1.0, and SCC*mec* elements were identified in sequenced *S. aureus* isolates by SCCmecFinder 1.2. All analyses were performed using default parameters. 

For virulence factor identification, the *S. aureus* genomes were interrogated for a pool of genes, including those reported for staphylococci (VirulenceFinder database), and determined using a database built for this study with CLC genomics workbench 10.01 tools (QIAGEN Aarhus, Denmark). 

#### 4.4.4. Data Availability

The *S. aureus* O20 and *S. aureus* D2 whole-genome shotgun (WGS) projects have been deposited at DDBJ/ENA/GenBank under the accession numbers JAHUTU000000000 (sequences JAHUTU010000001–JAHUTU010000273) and JAHUTV000000000 (sequences JAHUTV010000001–JAHUTV010000212), respectively.

### 4.5. Biofilm-Related Genes

DNA was extracted from fresh cultures as previously described [18]. Detection of 13 biofilm-related genes including, *icaA*, *icaB*, *icaC*, *icaD* (intercellular adhesion gene A, B, C and D, respectively), *fnbA* and *fnbB* (encoding fibronectin-binding protein A and B), *clfA* and *clfB* (encoding clumping factors A and B), *cna* (encoding collagen-binding protein), *eno* (encoding laminin-binding protein), *ebpS* (encoding elastin-binding protein), *fib* (encoding fibrinogen-binding protein) and *bbp* (encoding bone sialoprotein-binding protein) was performed by PCR using specific primers and conditions previously described [71].

### 4.6. Effect of Antibiotics on 24-h-Old Biofilm Biomass

The 18 strains analyzed by CSLM were used in this experiment. Biofilm formation was carried out as described above. After obtaining 24-h-old biofilms, the medium was removed carefully, and the culture medium was replaced by 200 µL of TSB solution with erythromycin (Sigma-Aldrich, Saint Louis, MO, USA), ciprofloxacin (Sigma-Aldrich, Saint Louis, MO, USA) or tetracycline (Sigma-Aldrich, Saint Louis, MO, USA) and incubated at 37 °C for 24 h without shaking. The concentrations used for each antibiotic correspond to the peak serum concentration, with 10.0 mg/L for erythromycin, 4.5 mg/L for ciprofloxacin and 16.0 mg/L for tetracycline [72,73,74]. After incubation with antimicrobial agents, biofilm biomass was quantified using the CV staining method as described in Section 4.2. All experiments had seven technical replicates and were performed in triplicate.

#### Effect of Antibiotics on Metabolic Activity

To determine the effect of antimicrobial agents on metabolic activity, after the incubation period with antimicrobial agents, biofilms were quantified using the XTT colorimetric method, as previously described by Logu et al. [75]. Biofilms were washed with 200 µL of 0.9% (*w*/*v*) NaCl solution and 200 µL of a solution containing 250 µg/mL of XTT (2,3-bis-(2-methoxy-4-nitro-5-sulfophenyl)-2H-tetrazolium-5-carboxanilide) (Panreac Applichem, Barcelona, Spain) and 25 µg of phenazine methosulfate (MSF) (Acros Organics, Geel, Belgium) were added. The plates were incubated in the dark for 3 h at 37 °C and, after incubation, 150 µL of the solution from each well was transferred to 1.5 mL Eppendorf tubes and centrifuged for 5 min at 10,000 rpm. Then, 100 µL of supernatant was collected, transferred to a new microtiter plate, and 100 µL of sterile ultrapure water was added to each well. Absorbance was measured at 490 nm. All experiments were performed in triplicate with two technical replicates.

### 4.7. Statistical Analysis

Statistical analyses were performed using IBM SPSS Statistics for Mac, Version 26.0. (IBM Corp., Armonk, New York, NY, USA) and GraphPad Prism Version 8.0.2. (GraphPAD Software Inc., San Diego, CA, USA) to compare the biofilm formation capacity of clinical isolates from different infections. Results were expressed as mean values and standard deviation. The level of significance was determined using one-way ANOVA with Tukey’s multiple comparison test. *p* ≤ 0.05 was considered significant.

## 5. Conclusions

Our results indicate that MRSA strains, independently of the type of infection, are biofilm producers. Interestingly, strains from osteomyelitis had a lower biofilm formation capacity and lower prevalence of antimicrobial resistance genes than MRSA from bacteremia and diabetic foot ulcers, at least under our tested conditions. *ica* genes were the most frequently detected genes among all isolates, demonstrated to be significant in biofilm formation. Furthermore, we also showed that the difference in the presence of just one biofilm-related gene may promote higher or lower biofilm formation. Understanding the ability of MRSA strains from different types of infections to form biofilms and the mechanisms underlying biofilm production is the first step towards a possible solution for biofilm-related infections.

## Figures and Tables

**Table 1:** Genome analysis of MRSA clinical strains.

Strain	Contigs	Contig Length (bp)	N50 (bp)	Total Assembly Length (bp)	Minimum Coverage	Mean Coverage	GC Content (%)
O20	273	200 to 127,245	26.219	2,795,149	10.2x	57.3x	32.7
D2	212	203 to 137,189	36.182	2,788,919	10.5x	63.4x	32.7

**Table 2:** General features of MRSA O20 and D2 clinical strains. PathogenFinder 1.1, ResFinder 2.1 (90% identity and 40% minimum length) and PlasmidFinder 1.3 (<98% homology) were used to estimate the number of pathogenicity determinants, genes and plasmids, respectively, within the genome. ISsaga: Semi-Automatic Insertion Sequences Annotation.

		O20	D2
**MLST**		ST22 (CC22)	ST105 (CC5)
**SCC*mec***		SCC*mec* type IV(2B)	SCC*mec* type II(2A)
***spa* typing**		t6966	t535
***agr* typing**		*agr* IV	*agr* II
**Antimicrobial resistance genes**		*erm(C)*, *blaZ*, *mecA* *gyrA (S85P)*	*erm(A)-type*, *aadD-type*, *ant(9)-Ia*, *blaZ*, *mecA-type* *gyrA (S84L)*, *grlA (S80Y)*, *grlA (E84G)*
**Virulence**	Adherence	*bbp, ebp, eno, fnbA, icaACDR, srtB*	*bbp, ebp, eno, fnbA*, *icaABCDR*, *sdrE*, *srtB*
	Exoenzymes	*adsA, aur, chp, geh, lip, sak, sspABC*	*adsA*, *aur*, *chp*, *geh*, *lip*, *sak*, *sspABC*
	Host Immune Evasion	*cap8, sbi, scn*	*cap8A*, *sbi*, *scn*
	Iron uptake and metabolism	*isdABCDEFG*	*isdABCDEFG*
	Toxins and secretion machinery	*cidA, esaAB, essAB, esxA, hlb, hld, hlgABC, hly/hla, hysA, pvl, sec, seg, sei, sel, sem, sen, seo*	*cidA, esaABDEG1G9*, *essABC*, *esxABCD*, *hlb*, *hld*, *hlgABC*, *hly/hla*, *hysA*, *lukD*, *pvl, sed*
**Plasmid type**		repL (rep10)	repA_N (rep20), rep1-type (rep22)
**Pathogen** (Probability of being a human pathogen)		97.8%	98.3%
**ISsaga**		15 ORFs related to IS(s) were found in these replicons: 5 Putative complete ORFs 6 Putative partial ORFs 4 Uncategorized ORFs	11 ORFs related to IS(s) were found in these replicons: 4 Putative complete ORFs 5 Putative partial ORFs 2 Uncategorized ORFs

**Table 3:** Biofilm-forming capacity and susceptibility to the tested antibiotics of the 18 selected strains.

Strain	% of Biofilm Formation	Phenotypic Antimicrobial Resistance
S1	67.7%	CIP
S6	99.2%	ERY, CIP
S7	69.8%	CIP
S8	64.7%	ERY CIP
S10	82.6%	ERY, CIP
S12	78.9%	CIP
D2	105.1%	ERY, CIP
D5	94.3%	Susceptible
D6	73%	Susceptible
D7	65.2%	CIP
D15	84.8%	ERY, CIP
D26	76.4%	ERY, CIP, TET
O11	37.4%	CIP
O19	51.6%	ERY, TET
O20	31.9%	ERY, CIP, TET
O25	56.9%	ERY, CIP
O26	60.8%	Susceptible
O39	66.5%	CIP
